# Memory capacity of networks with stochastic binary synapses

**DOI:** 10.1186/1471-2202-14-S1-P43

**Published:** 2013-07-08

**Authors:** Alexis Dubreuil, Yali Amit, Nicolas Brunel

**Affiliations:** 1 Department of Neurobiology, University of Chicago, Chicago, IL 60637, USA; 2 Department of Statistics, University of Chicago, Chicago, IL 60637, USA; 3 Brain Physiology lab, CNRS & Université Paris Descartes, Paris, France

## 

Lesion studies suggest that episodic memories are initially stored in the hippocampus, but are then transferred to cortex where a long-term memory trace is stored. This suggests that in the hippocampus, memories have to be acquired in one shot, while in cortex, they are acquired slowly over multiple repetitions. In the present work, we study the memory capacity of networks that have to acquire memories either in one shot, or through multiple presentations.

To allow for analytical tractability we used networks of N binary neurons connected with binary synapses. Stochastic Hebbian-type synaptic plasticity occurs upon pattern presentation, and two parameters q+ and q- control the amounts of potentiation and depression, as in [1,2]. Stored patterns are random, sparse and uncorrelated, and are characterized by a coding level f that scales as ln N/N. In the hippocampal-like condition (H), the network is presented a flow of patterns that appear only once. In the cortical-like condition (C), a set of P patterns is repeatedly presented. The storage capacity was defined as the maximal number of patterns that can be stabilized as fixed-point attractors of the network. We computed this storage capacity of networks under (H) or (C) conditions, allowing us to find parameters (f, q+, q-, and the threshold of neurons) that optimize storage. In both cases, the maximal number of stored patterns scales as N^2/(ln N)^2, with a prefactor that we compute explicitly. The capacity can also be expressed in terms of information stored per synapse (in bits) Imax, which is finite in the large N limit. Under condition (H), the model is the one studied in [1], we find Imax = 0.053 bits/synapse in the large N limit. This number is lower than the capacity for the Willshaw model (0.264)[3,4] and the Gardner bound (0.29)[5,6]. This loss in capacity is the price to pay for the palimpsest property. In the cortical-like condition, the capacity was computed in the slow learning regime (q+, q- small, cf [2]) and we found that information capacity is optimized if the effects of depression are minimal. In this condition, the information capacity is shown to be equal to the one of the Willshaw model, and is therefore very close to the Gardner bound. We then proceed to study finite-size effects, and show that these effects are of order (ln(ln N)/ln N)^1/2, and are therefore very large even in networks of realistic sizes (e.g. 10^5). We find that the capacity for networks of sizes of order 10^4-10^6 are only 50-60% the capacity in the large N limit. Analytical results are shown to be in very good agreement with simulations up to sizes N = 40,000.

Overall, we find that the capacity of networks of binary neurons and binary synapses with the simplest stochastic learning rule is surprisingly close to the theoretical capacity limits.
